# MicroRNA expression analysis in endometriotic serum treated mesenchymal stem cells

**DOI:** 10.17179/excli2017-101

**Published:** 2017-06-14

**Authors:** Mazen Abdel-Rasheed, Ghada Nour Eldeen, Marwa Mahmoud, Mahmoud ElHefnawi, Nourhan Abu-Shahba, Mohamed Reda, Khaled Elsetohy, Michael Nabil, Amr Elnoury, Tamer Taha, Osama Azmy

**Affiliations:** 1 Department of Reproductive Health Research, National Research Centre, Cairo, Egypt; 2 Stem Cell Research group, Medical Research Centre of Excellence, National Research Centre, Cairo, Egypt; 3 Department of Molecular Genetics and Enzymology, National Research Centre, Cairo, Egypt; 4 Department of Medical Molecular Genetics, National Research Centre, Cairo, Egypt; 5 Biomedical Informatics and Chemo-informatics group, Informatics and Systems Department, National Research Centre, Cairo, Egypt; 6 Department of Obstetrics and Gynecology, Faculty of Medicine, Cairo University, Cairo, Egypt; 7 Department of Molecular Genetics, CliniLab, Cairo, Egypt; 8 Department of Medical Applications of Laser, National Institute of Laser Enhanced Sciences, Cairo University, Cairo, Egypt

**Keywords:** endometriosis, mesenchymal stem cells, miRNA expression, differentiation

## Abstract

Endometriosis is defined by presence of endometrial-like-tissue outside the uterus. Recently, ectopic endometriotic lesions have been suggested to originate by abnormal differentiation of endometrial mesenchymal stem cells (eMSCs). MicroRNAs (miRNAs) play an important role in the pathophysiology of endometriosis. Through a PCR array approach, we aimed to assess the differential expression of microRNAs in human eMSC treated in culture with sera derived from women with severe endometriosis. Sera were collected from five patients with severe endometriosis and three control women and added individually in the culture medium to conduct experimental and control eMSC sets, respectively. Regular microscopic follow-up for cell morphology was performed. SYBR Green based real-time PCR array was used to assess the expression of 84 miRNAs. Bioinformatics analysis was done to predict the target genes of the significantly dysregulated miRNAs and their enriched biological processes and pathways. Thirty-two miRNAs were found significantly dysregulated in experimental cultures. Functional enrichment analysis revealed several endometriosis associated biological processes and pathways were enriched by target genes of these miRNAs. In conclusion, treatment of human eMSCs with sera of severe endometriosis cases affects the expression of certain miRNAs and their target genes. This may result in altering cell functions and consequently, endometriosis development.

## Introduction

Endometriosis is an inflammatory disorder, characterized by the presence of endometrial-like-tissue outside the uterus in pelvic or extra pelvic locations. It affects about 6 to 10 % of reproductive aged-women and is associated with chronic pelvic pain and infertility (Giudice, 2010[17]). Although an endometriotic implant is a benign tissue, it shares certain characteristics with cancer such as cell migration, adhesion, invasion, matrix remodeling, proliferation, survival, angiogenesis promotion, and escaping immune surveillance (Burney and Giudice, 2012[7]). Several theories have been introduced to explain endometriosis etiology (Maruyama, 2014[35]), however, they are still not fully confirmed (Sourial and Tempest, 2014[50])*.* Improving knowledge on endometriosis pathogenesis may assist in identifying novel targets for formulating more effective therapies (Sourial and Tempest, 2014[50]). 

Since the discovery of endometrial stem/progenitor cells, accumulating evidences have been provided for the involvement of these undifferentiated cells in endometriosis development (Sasson and Taylor, 2008[49]).

Among the endometrial progenitors that have been identified are mesenchymal stem/stromal cells (MSCs) (Gargett et al., 2009[14]). MSCs are clonogenic fibroblast like cells that have the potential to self-renew and differentiate into multiple lineages (Gargett et al., 2016[13]). A number of investigators revealed that ectopic implants-derived MSCs had enhanced proliferation, migration, invasion and angiogenic abilities, over eutopic counterparts, proposing that endometrial MSCs may be one of stem cells which participate in endometriosis progression (Kao et al., 2011[30]; Hsu et al., 2014[23]). 

MicroRNAs (miRNAs) act as post-transcriptional gene expression regulators (Macfarlane and Murphy, 2010[33]). miRNAs play essential roles in nearly all biological and pathological processes in the human body (Ha and Kim, 2014[20]). Among the various pathological conditions in which miRNAs take part, are gynecological and fertility disorders (Gilabert-Estelles et al., 2012[15]). In this context, several studies have revealed that aberrant expression of miRNAs has a direct potential role in the pathogenesis of endometriosis (Pan et al., 2007[43]; Ohlsson Teague et al., 2009[40]; Filigheddu et al., 2010[11]; Hawkins et al., 2011[22]). miRNAs are key candidates in regulating cell fate through controlling different cellular biological processes as proliferation, apoptosis and differentiation. Accordingly, miRNAs were found to be the main actors in stem cell development and commitment (Mathieu and Ruohola-Baker, 2013[36]). 

Earlier, our group demonstrated that culture of human MSCs (hMSCs) with serum derived from moderate and severe endometriosis cases had induced morphological and molecular changes providing evidence that serum of women with endometriosis harbors a possible endometriosis inducing factor(s) (EIF) that enables the MSCs to acquire the phenotype of endometrial-like-cells *in vitro* (Rasheed et al., 2010[45], Azmy et al., 2014[3]).

Later on, by assessing the differential expression of 84 miRNAs in the sera of severe endometriosis cases, we suggested that miR-130a may be EIF mediates the trans-differentiation of MSCs into endometrial-like cells, in addition to regulating gene expression in several endometriosis related biological processes and cell functions (Azmy and Elgarf, 2012[4]; Azmy et al., 2014[3]).

## Materials and Methods

### Study population

This study represents an experimental prospective case-control pilot study, including eight women subjects. It was approved by the Medical Research Ethics Committee of the National Research Centre, Cairo, Egypt, under registration number 12-002. Written informed consents were obtained from all participants to collect samples as well as to publish the results. 

The samples were recruited from the Obstetrics and Gynecology Department, Faculty of Medicine, Cairo University. The enrolled women in the study met the following criteria; they suffered from infertility and/or pain and underwent laparoscopy for diagnosis, they did not receive any hormonal therapy 6 months prior to the time of sample collection, they did not have a history of blood malignancies, chronic or immunological diseases. Of the eight participants, five had severe endometriosis (the experimental group) and three were endometriosis free (the control group). The severity of the disease was clinically identified according to the revised American Society of Reproductive Medicine staging system (rASRM, 1997[46]). Endometriosis laparoscopic diagnosis was confirmed by histopathological examination, while, the laparoscopy inspection in control subjects showed that they were clearly free from any endometriotic lesions. 

### Serum collection

Peripheral blood samples were collected from patients (n=5), and control (n=3). Whole blood was drained into vacutainer without anticoagulants and allowed to clot overnight at 4 °C. Serum was aliquoted and separated by centrifugation at 2.000 rpm for 15 min. Subsequently the supernatant was aliquoted and frozen at -20 °C. 

### Tissue collection

Specimens of endometrial tissues were collected under sterile conditions from women suffering from dysfunctional uterine bleeding undergoing curettage as a treatment of choice in severe cases, in the operating theatre room. Part of the endometrial tissues were sent to pathological examination and the rest of specimens were immediately placed in DMEM low glucose media containing antibiotic/antifungal mix to be further processed for mesenchymal stromal cell isolation within two hours of procurement. Endometrial tissues with pathological conditions were discarded.

### Isolation and culture of endometrial mesenchymal stromal cells 

As previously reported (Kao et al., 2011[30]), the collected endometrial tissue samples were enzymatically digested to drive the tissue mononuclear cells which were cultured in DMEM low glucose medium (Lonza) supplemented with 10 % FBS, 100 units/ml penicillin (Gibco), 100 μg/ml streptomycin (Pen-Strep, Lonza) and 2 mM/L glutamax (Gibco). The cultures were then incubated in humidified atmosphere with 5 % CO_2_ concentration in CO_2 _incubator (Sartorius Stedim Biotech, GmbH, Germany). Media exchange was done every 2-3 days. After attaining confluence, the cultures underwent subsequent passaging until passage 3.

### Cell culture treatment

At the third passage, thirty percent confluent eMSC cultures were challenged with the previously collected sera. To conduct experimental and control cultures, five endometriotic and three control serum samples were added, separately, to the regular growth medium with a final serum concentration (5 %) respectively. An experimental or a control culture was fed once per 2 weeks with the media containing the corresponding serum sample for a period of 8 weeks. 

### Microscopic follow up and photo-documentation

The morphology of cultured MSCs was examined periodically under inverted microscope (Nikon eclipse TS 100, Japan) and was photographed using digital eyepiece camera (Premiere, MA 88-500).

### MicroRNAs expression analysis

#### RNA extraction and cDNA synthesis

Total RNA was isolated from cell cultures with the mirVana miRNA Isolation Kit (Ambion, USA) according to the manufacturer's protocol. RNA purity and concentration were measured using a NanoDrop ND-2000 spectrophotometer (Thermo Scientific, USA). Then, RNA was reverse transcribed to cDNA using a Superscript II First Strand Synthesis System (Qiagen). Each reverse transcriptase (RT) reaction contained 125-250 ng of RNA sample, 2 µl of 10x miScript Nucleic Mix, 10x miScript Reverse Transcriptase mix, and 4 µl 5× miScript HiSpec Buffer (all included in First-strand cDNA synthesis kit), with total volume of 20 µl. Reactions were incubated in a thermal cycler (Applied Biosystem) for 60 minutes at 37° C, followed by a heat-inactivation step for 5 minutes at 95° C and held at 4° C.

#### MicroRNA real time polymerase chain reaction

The expression levels of a panel of 84 miRNAs were assessed using an SYBR Green-based miScript miRNA PCR array (Human miFinder miRNA PCR Array: MIHS-001Z, Qiagen). The analyzed miRNAs are a group of 84 most abundantly expressed and well characterized miRNAs in miRBase (http://www.miRBase.org). Three control sets were present in this panel; the first contained six miRNAs (snoRNA/snRNA) whose average of readings enables normalization of the array data using the relative quantification method, the second was for assessing the performance of reverse transcription reaction and the third was for assuring PCR performance. 

Reaction mixture for pathway-focused miScript miRNA PCR array was prepared according to manufacturer's instructions (Qiagen). Quantitative real-time PCR (qPCR) analysis was performed in an ABI 7500 Detection System (Applied Biosystems). The thermal conditions for qPCR were as follows: initial activation step at 95 °C for 15 minutes, 50 cycles of denaturation at 94 °C for 15 seconds followed by an annealing step at 55 °C for 30 seconds, then extension step at 70 °C for 30 seconds. Relative miRNA expression was calculated using the 2^-ΔΔCt^ method (Livak and Schmittgen, 2001[32]). All experiments were performed on two different endometrial MSC lines.

### Statistical analysis

Data were expressed as mean ± standard error. Statistical differences between means of experimental and control groups were analyzed using unpaired Student's t-test. P values less than 0.05 was considered statistically significant. Statistical analysis was carried out using the SPSS 16.0 software (IBM, New York, USA).

### Bioinformatics analysis

**Figure 1:**
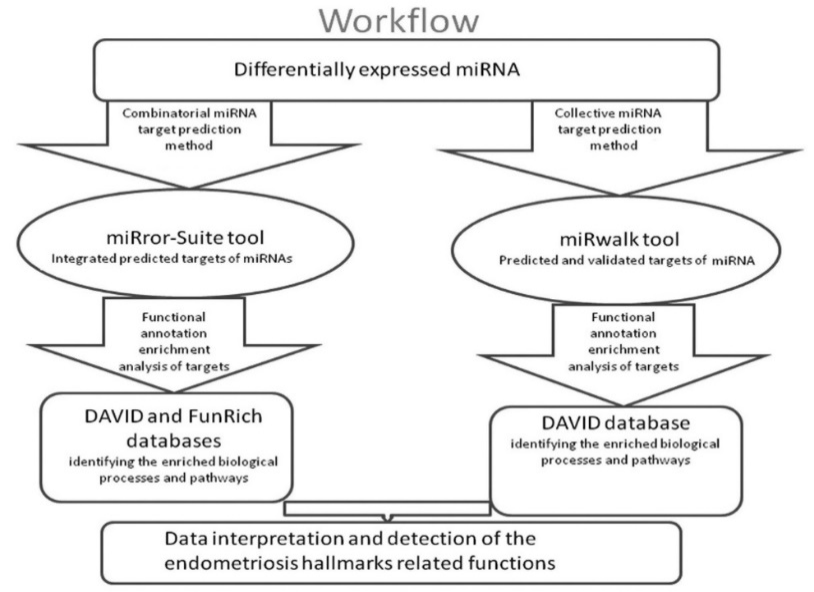
A flow chart illustrating our computational miRNA target prediction and functional analysis steps

Computational prediction of miRNA targets is an important step for exploring the miRNA-mRNA interactions. We identified the *in silico* predicted and validated targets of the significantly differentially expressed miRNAs and performed the functional enrichment analysis of these targets using different bioinformatics tools. Our workflow is shown in Figure 1(Fig. 1).

### MicroRNA computational target analysis

MicroRNA target analysis was done by two parallel protocols: combinatorial miRNA target analysis; where target prediction was performed for a whole defined miRNA set, and collective miRNA target analysis, where target analysis was performed for each miRNA in a defined set followed by integrating the obtained targets for functional and pathway enrichment.

*Combinatorial Method:* Combinatorial analysis was performed using the bioinformatics tool; miRror Suite 2.0 [http://www.proto.cs.huji.ac.il/mirror/]. 

*Collective Method:* Predicted and validated target genes of an individual miRNA of our differentially expressed miRNAs were derived using miRWalk 2.0 server [http://mirwalk.uni-hd.de/]. 

### Functional annotation enrichment analysis

For both combinatorial and collective methods, the functions of the obtained miRNA target genes and the pathways in which they could be involved were analyzed using the DAVID server (Database for Annotation, Visualization and Integrated Discovery), [https://david.ncifcrf.gov]. It identifies the enriched gene ontology terms and pathways from KEGG, Biocarta, Panther, Reactome, and others to help finding out the most relevant functions associated with a given gene list (Dennis et al., 2003[9], Huang da et al., 2009[24]). For the combinatorial method, target genes were also submitted to FunRich 2.1.1 (Functional enrichment analysis tool for biological processes and pathways analysis) [http://funrich.org/index.html]. It is standalone functional enrichment analysis software that permits offline data analysis on the user's desktop (Pathan et al., 2015[44]). The key enriched pathways and gene ontology annotations, which were suggested to be associated with endometriosis, were then investigated.

## Results

In the present work, stroma of endometriosis free endometria were used as a source for MSCs. A sub-confluent eMSC culture at passage (3) was challenged with either an endometriotic or a non-endometriotic serum. Two main eMSC culture sets were established: experimental set; endometriotic serum treated cultures (n=5) and the control set; non-endometriotic serum treated cultures (n=3). The two sets were studied in terms of morphological changes and expression profile of 84 miRNAs using a SYBR Green-based real-time PCR array. 

### Microscopic follow up for the morphological characteristics of eMSC cultures during the expansion/proliferation phase

**Figure 2:**
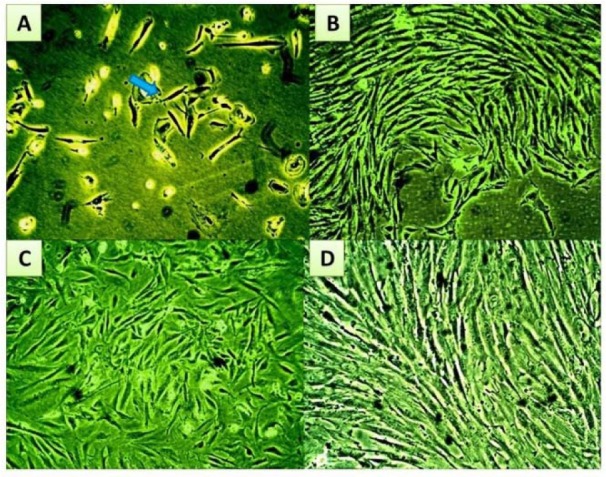
Microscopic follow-up for morphological characteristics and proliferation performance of eMSC cultures during expansion phase (A) Shows a small colony of short spindle cells at day 7 of primary culture, which by continuous growth, increased in size to fuse with an adjacent colony, shown in (B), forming a confluent monolayer of fibroblastic eMSCs at the end of P0 (C). (D, E, and F) Show confluent monolayer of eMSCs at the end of passage (2). The arrow points to the fibroblastic morphology. Magnification power X40

Endometrial mesenchymal stromal cells were isolated and cultured as described in materials and methods. The morphology of eMSCs that were cultured in the regular growth medium, before serum challenge, is shown in Figure 2(Fig. 2). Plastic adherent short spindle fibroblast like cells appeared in the culture flasks after a few days of incubation. After a week, the cells gradually grew to form small colonies (Figure 2A(Fig. 2)). As growth continued, colonies gradually expanded in size and the adjacent ones became interconnected (Figure 2B(Fig. 2)). These primary cells formed a confluent monolayer after 10-12 days of initial plating (Figure 2C(Fig. 2)). By subsequent passaging, homogenous confluent layer of elongated spindle fibroblast like cells predominated (Figure 2D(Fig. 2)).

### Morphological and growth characteristics of eMSCs during the serum challenge phase

**Figure 3:**
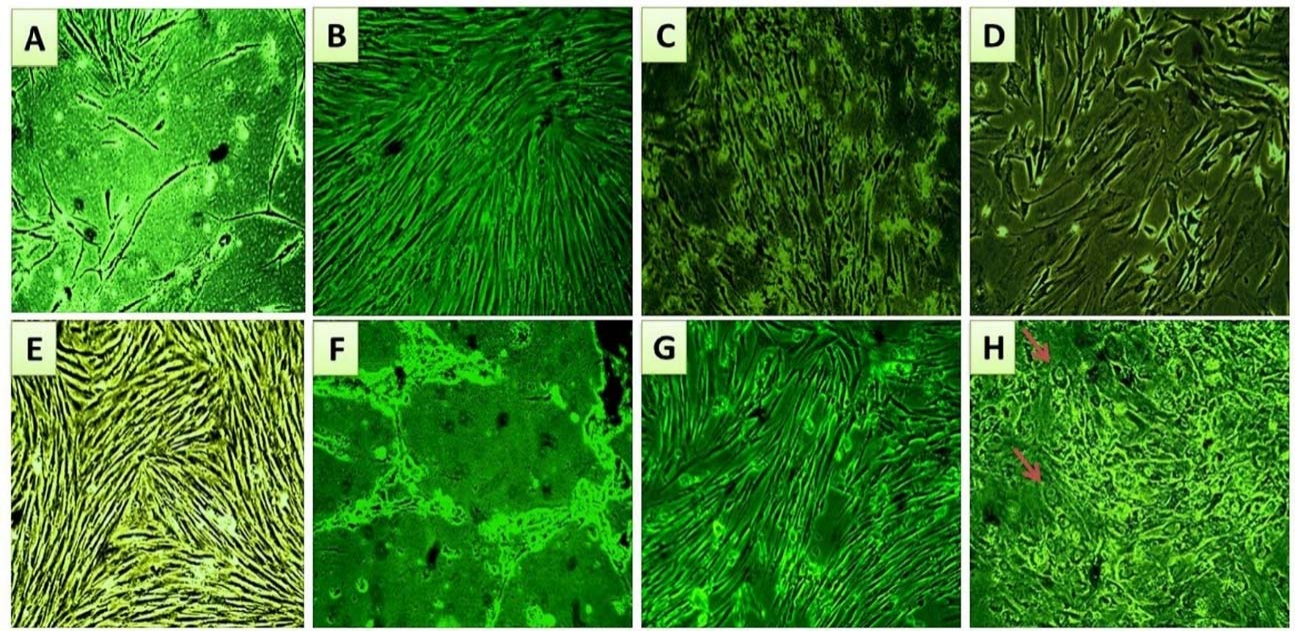
Microscopic follow-up for morphological characteristics and proliferation performance of eMSC cultures during serum challenge phase. (A) Shows eMSC culture at passage 3 just before serum application. (B) Shows control serum treated eMSC culture with fibroblastic morphology and enhanced proliferation at the second week of serum incubation. (C and D) are representative photos for the experimental cultures within the first two weeks of serum addition. They show relatively maintained fibroblastic morphology and cell proliferation. (E) Shows control culture with typical spindle morphology and maintained proliferation capacity at the sixth week of serum addition. (F) Shows an obvious decline in cell growth and proliferation accompanied with cell shrinkage in some experimental eMSC cultures, at week 6 of endometriotic serum challenge. (G) Shows lesser decrease in cell proliferation and predominant fibroblastic morphology in other eMSC cultures at week 6 of endometriotic serum addition, however, irregular morphologies appeared occasionally in some fields, as indicated by arrows (H). Magnification power X40

It was observed that cell proliferation and morphology in the experimental cultures, treated with endometriotic sera, had changed gradually by time, compared to those treated with non-endometriotic sera (Figure 3(Fig. 3)). Within the first two weeks of challenge, the experimental cultures showed maintained proliferation and fibroblastic morphology (Figure 3C and D(Fig. 3)). At later weeks, various morphological changes appeared associated with moderate to high decrease in cell growth and proliferation among experimental cultures compared to control cultures (Figure 3E(Fig. 3)). Shrinkage of fibroblastic like- cells of some experimental cultures was observed (Figure 3F(Fig. 3)), while other cultures kept the fibroblastic morphology (Figure 3G(Fig. 3)); with occasional appearance of irregular cell morphologies in some areas (Figure 3H(Fig. 3)).

### MicroRNA expression analysis

Using miFinder miRNA PCR Array platform, the expression profile of 84 miRNAs was assessed for the control and experimental cultures. Data were presented as fold-change relative to the control group. The average cycle threshold of six miRNAs; hsa-mir-101-3p, 122-5p, 141-3p, 142-5p, 302b-3p, and 9-5p were greater than the defined cut-off value (default 40) in the control group, making their fold changes uninterpretable. Therefore, they were excluded and 78 miRNAs only were analyzed. According to the statistical analysis, 32 differentially expressed miRNAs were found significant in the experimental cultures of all samples. 

### Bioinformatics analysis

Bioinformatics analysis was performed for the significantly dysregulated miRNAs obtained by statistical analysis.

In the combinatorial method, the 32 differentially expressed miRNAs submitted to the miRror Suite combinatorial miRNA target prediction tool resulted with 530 target genes, with cut off p-value 0.05. In the collective method, the total number of integrated predicted and validated targets obtained by miRWalk tool for the 32 miRNAs was 6594 target genes. These target genes were submitted for subsequent functional enrichment analysis. 

**Figure 4:**
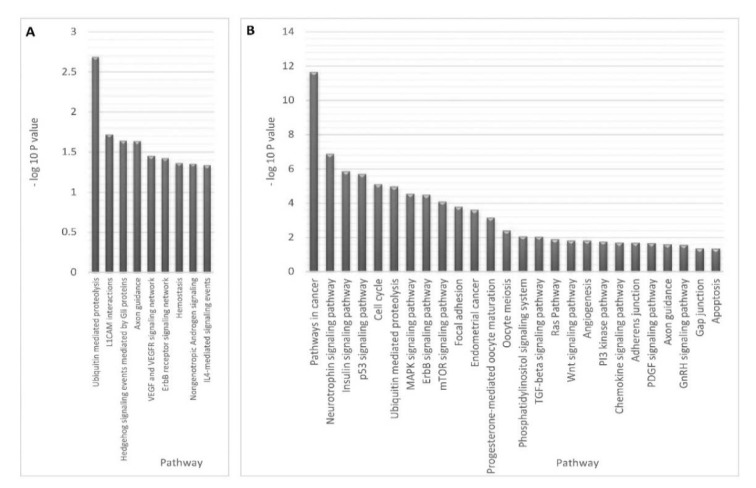
Enriched endometriosis pathways versus -Log 10 P value for miRNA target genes of differentially expressed miRNAs. The figure shows the functional pathways that are significantly enriched in response to miRNA dysregulation in endometriosis obtained by combinatorial method (P value < 0.05) (A) and collective method (Benjamini < 0.05) (B).

Functional enrichment analysis of the target genes obtained from the combinatorial miRNA target prediction analysis was done by submitting those predicted genes to DAVID and FunRich enrichment analysis tools for identifying the enriched biological processes and pathways (Table 1(Tab. 1)). Among the biological processes which have shown statistical significance (P-value <0.05), there were several processes known to be involved in endometriosis such as small GTPase mediated signal transduction including Ras and Rho protein signal transductions**, **ubiquitin cycle**, **histone modification, progesterone receptor signaling pathway and cellular morphogenesis during differentiation (Table 2(Tab. 2)). Also, there were various endometriosis related pathways that showed significant enrichment (P-value <0.05) for the 32 miRNA targets such as axon guidance, L1CAM interactions**, **hedgehog signaling events mediated by Gli proteins, VEGF and VEGFR signaling network, IL4-mediated signaling events, and ErbB receptor signaling network (Figure 4A(Fig. 4)).

Meanwhile, the total target genes of the 32 miRNAs obtained from the collective method underwent functional enrichment analysis for identifying the enriched biological processes and pathways using DAVID database. This analysis resulted in a plenty of enriched endometriosis related biological processes and pathways with high enrichment statistical significance (Benjamini value < 0.05). These biological processes involve nearly most of the key processes which are involved in endometriosis such as cell cycle, apoptosis, cell migration, positive regulation of cell differentiation and proliferation, epithelium development, vasculature development**, **angiogenesis, cell migration, regulation of cell motion, regulation of cell adhesion, positive regulation of cell communication, cellular response to stress, response to hypoxia, MAPKKK cascade and regulation of mitogen activated protein kinase (MAPK) activity, epidermal growth factor receptor (EGFR) signaling, reproductive developmental process, response to steroid hormone stimulus, and others (Table 2(Tab. 2)).

Moreover, many significantly enriched pathways that are possibly involved in endometriosis were identified through our analysis using the gene list of the collective method. These pathways include pathways in cancer, p53 signaling pathway, neurotrophin signaling pathway, MAPK signaling pathway, ErbB signaling pathway, mTOR signaling pathway, focal adhesion, endometrial cancer, TGF-β signaling pathway, Ras Pathway, Wnt signaling pathway, PI3K pathway*, *PDGF signaling pathway, GnRH signaling pathway, adherens junction, gap junction, axon guidance and others (Figure 4B(Fig. 4)).

The common enriched endometriosis related biological processes between the two methods were small GTPase mediated signal transduction, Ras protein signal transduction, ubiquitin cycle, and cellular morphogenesis during differentiation. Whereas the common enriched pathways between both methods were axon guidance, ErbB signaling, and ubiquitin mediated proteolysis.

## Discussion

Endometriosis is a gynecological disease that causes socioeconomic burden compromising women's quality of life and is a common cause of infertility. In the present study, we investigated the effect of the stressful pathogenic condition of endometriosis, represented in endometriotic serum, on human endometrial mesenchymal stromal/stem cells (eMSCs) with regard to their miRNA expression. This study design was based on our previous observation which revealed that serum of women with endometriosis possibly harbors endometriosis inducing factor(s) (EIF) that can lead to the transformation of MSCs into endometrial like cells and glands *in vitro* (Rasheed et al., 2010[45]; Azmy and Elgarf, 2012[4]; Azmy et al., 2014[3]). 

Treatment of human eMSC culture with the serum of severe endometriosis cases was found to affect the expression of certain miRNAs and in turn, their target genes that may play major roles in modulating cell functions and fate. We profiled the expression of 84 well-characterized miRNAs in control and endometriotic serum treated eMSCs using human miFinder miRNA PCR array (MIHS-001Z); a readymade miRNA panel designed to profile the expression of the most abundantly expressed and best characterized miRNAs in the miRBase database. The complex role of each of these miRNAs in this panel, in different diseases, is still not completely determined. Endometriotic serum treated eMSCs showed alterations in the expression of several analyzed miRNAs, thirty-two of which were significantly differentially expressed. The 32 significantly altered miRNAs were found to regulate 530 target genes derived by miRror Suite combinatorial miRNA target prediction tool and 6594 target genes derived by miRWalk tool. Functional analysis of both gene lists revealed that they enrich signaling pathways and biological processes, corroborate known endometriosis associations in the literature.

Human mesenchymal stem/stromal cells (hMSCs) are multipotent precursors, located throughout the adult body tissues, especially in those with stromal vascular fractions, and believed to be involved in maintenance and repair of tissues (Hass and Otte, 2012[21]). MSCs in culture exhibit a preferential ability to adhere to plastic surfaces facilitating their isolation (Jiang et al., 2002[28]). Depending on this characteristic and by enzymatic processing for normal (endometriosis free) endometria, endometrial hMSCs were isolated and propagated in the suitable growth conditions. Coping with previous studies (Kao et al., 2011[30]), cells displayed the typical fibroblast-like morphology and exerted high expansion potential. At early passages, cultures were challenged with either endometriotic or control serum conducting two culture sets; experimental and control. Morphological and proliferative alterations were specifically observed in cultures exposed to endometriotic serum, reflecting its possible impact on eMSC cultures. 

Among the potent epigenetic players that regulate most of the biological processes involved in endometriosis pathogenesis are miRNAs (Mari-Alexandre et al., 2016[34]). Moreover, miRNAs are key candidates in regulating stem cell fate through controlling different cellular actions as proliferation and differentiation (Mathieu and Ruohola-Baker, 2013[36]). In our study, among the 32 significantly dysregulated miRNAs, ten miRNAs; miRs-150, 23b, 106b, 17-5p, 20a, 29c, 125b, 143, 23a and 21, were found to be repeatedly reported as endometriosis related miRNAs (Wei et al., 2015[52]). Meanwhile, some of the remaining 22 aberrantly expressed miRNAs in our study were significantly dysregulated in endometriotic tissues in individual studies such as miRs- 19b, 22, 27b, 29a, 30a-5p, 30b, 30c, 103a, 191, 195 (Pan et al., 2007[43]), miR-424 (Ohlsson Teague et al., 2009[40]), miRs-186 and -93 (Filigheddu et al., 2010[11]). 

Supporting to the endometriotic miRNA dysregulation that is reported here, several studies have introduced a number of miRNAs that were aberrantly expressed in stromal cells derived from endometriotic tissues, compared to those derived from eutopic or normal endometria (Lin et al., 2012[31]; Abe et al., 2013[1]; Hsu et al., 2014[23]; Okamoto et al., 2015[41]). However some of these reports investigated miRNAs that were not enrolled in our panel such as miR-199a-5p (Hsu et al., 2014[23]), and miR-210 (Okamoto et al., 2015[41]), others reported endometriosis related dysregulation in some of miRNAs that were significantly dysregulated in the present work such as miR-20a (Lin et al., 2012[31]), miR-424 and miR-181a (Abe et al., 2013[1]). 

Herein, 29 miRNAs out of our 32 differentially dysregulated miRNAs were overexpressed, while three (miR-144-3p, 150-5p, 96-5p) were down-expressed. Such expression direction for a number of miRNAs was different from some literature data. For example, hsa-mir-20a was found to be downregulated in endometriotic tissues, compared with normal or eutopic endometrium (Pan et al., 2007[43]; Ohlsson Teague et al., 2009[40]; Filigheddu et al., 2010[11]), however, coping with our study, it was reported upregulated in endometriotic stromal cell cultures (Lin et al., 2012[31]). This might be contributed to our different approach, where we used endometriotic serum challenged eMSCs and not an endometriotic tissue as in most literature studies. 

In our study, we used two *in silico* analysis tools for the differentially expressed miRNAs, obtained from the PCR array performed. These tools were miRror tool, which is a combinatorial miRNA target prediction algorithm, and miRWalk tool, which provides both predicted and experimentally validated miRNA targets. The main purpose for using both tools was to have an integrative view of combinatorial and individual microRNAs interactions and hence functions and pathways. Within each tool, we used the intersections of diverse prediction tools for microRNA targets to get the most assured targets and minimize false positives so that we can depend on these results to be used in functional enrichment analysis. 

Our obtained functional enrichment analysis results agree with several previous studies which have performed enrichment analysis for differentially expressed miRNAs in endometriotic samples (Ohlsson Teague et al., 2009[40]; Filigheddu et al., 2010[11]; Hawkins et al., 2011[22]; Aznaurova et al., 2014[5]; Wei et al., 2015[52]). The obtained biological processes and pathways belong nearly to most of the key cellular functions that take part in endometriosis pathogenesis. Among these processes are cell cycle and its related processes such as apoptosis, cell death and proliferation; cell differentiation and epithelium development (Ohlsson Teague et al., 2009[40]; Giudice et al., 2012[16]; Aznaurova et al., 2014[5]); angiogenesis and related processes including vasculature development (Rocha et al., 2013[47]), VEGF and VEGFR signaling network (Donnez et al., 1998[10]); cell migration and regulation of cell motion (Witz, 2003[55]); cell adhesion and related actions such as regulation of cell adhesion (Witz, 2003[55]), L1CAM interactions (Finas et al., 2008[12]), positive regulation of cell communication (Zhang et al., 2015[56]), focal adhesion (Mu et al., 2008[39]), adherens junction and gap junction (Giudice et al., 2012[16]); cellular response including response to stress, response to hypoxia (Gupta et al., 2006[19]) and response to steroid hormone stimulus (Giudice et al., 2012[16]).

In addition, several signaling pathways that play crucial roles in most endometriosis cellular processes were found to be enriched in our analysis, such as MAPK signaling pathway, MAPKKK cascade, regulation of MAPK activity (Santulli et al., 2015[48])*; *EGFR signaling (Huang and Yeh, 1994[25]; Ohlsson Teague et al., 2009[40]); mTOR signaling pathway (Choi et al., 2014[8]); TGF-β signaling pathway (Aznaurova et al., 2014[5]); Wnt signaling pathway (Matsuzaki et al., 2014[37]); PI3K pathway (Giudice et al., 2012[16]); PDGF signaling pathway (Aznaurova et al., 2014[5]); p53 signaling pathway (Giudice et al., 2012[16]), and hedgehog signaling events mediated by Gli proteins (Jeong, 2014[27]). Moreover, pathways in cancer and endometrial cancer (Giudice et al., 2012[16]), were among the obtained enriched pathways that are possibly involved in endometriosis as it was deduced that cancer related pathways were also found enriched in endometriosis (Wei et al., 2015[52]). Other endometriosis related pathways were enriched in our analysis as GnRH signaling pathway (Weng et al., 2014[53]), progesterone receptor signaling pathway (Wetendorf and DeMayo, 2014[54]), neurotrophin signaling pathway (Borghese et al., 2010[6]), histone modification (Monteiro et al., 2014[38]), and IL4-mediated signaling events (OuYang et al., 2008[42]). 

Importantly, our 2 methods of bioinformatics analysis (combinatorial and collective) revealed a group of endometriosis associated biological processes and pathways which were commonly enriched by both ways and consequently we considered them the most important endometriosis related functional events that take place in our serum treated cells. Among the commonly enriched endometriosis related biological processes were “small GTPases mediated signal transduction”, and “Ras (a GTPase) protein signal transduction”. Small GTPases are a large family of monomeric proteins that bind and hydrolyse GTP to GDP. They comprise several super families such as Ras, Rho and Rac (Goitre et al., 2014[18]). The Ras GTPases superfamily members are the most famous members of the small GTPases family. They act as regulators of cell growth, differentiation, and survival (Ohlsson Teague et al., 2009[40]). Ras network was found to be one of the essential molecular networks mediating the cellular and molecular crosstalk between endometrial and peritoneal tissues (Ohlsson Teague et al., 2009[40]). Therefore, these signal transduction processes play important roles in endometriosis development. In addition, ubiquitin cycle and ubiquitin mediated proteolysis were commonly obtained from our both methods of analysis. Previously, ubiquitin was found to be highly expressed in endometriotic cells where it was proposed to be responsible for the survival of ectopic stromal cells. It was deduced that its expression may contribute to reduced sensitivity of ectopic endometriotic cells to apoptosis and consequently the development of ectopic lesions (Ilad et al., 2004[26]). Another common biological process obtained from the 2 bioinformatics analysis methods was “cellular morphogenesis during differentiation”. Differentiation is one of the crucial processes through which stem cells act in the pathogenesis of endometriosis. Morphogenesis, which is a part of the differentiation process, is the mechanism through which tissues, such as vascular and glandular tissues, acquire their shapes and completely develop inside the endometriotic lesions (Sasson and Taylor, 2008[49]; Vasquez et al., 2016[51]).

**Figure 5:**
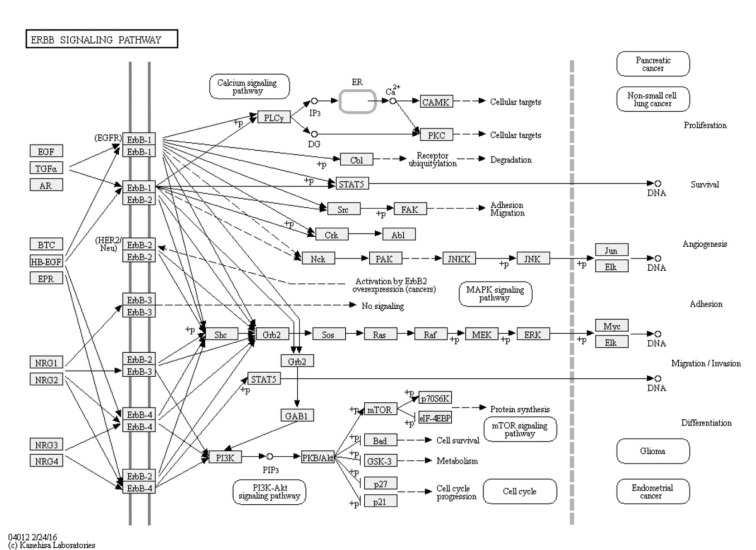
ErbB signaling pathway from Kyoto encyclopedia of genes and genomes. ErbB signaling pathway was predicted to be relevant to endometriosis. It is a key pathway that is involved in different essential endometriosis related cellular processes such as proliferation, differentiation, cell motility, migration, adhesion and survival (http://www.genome.jp/kegg-bin/show_pathway?hsa04012).

Moreover, two important endometriosis related pathways were commonly enriched by our both methods of analysis, which are ErbB signaling and axon guidance. ErbB signaling pathway has been detected previously as a significant endometriosis related pathway (Huang and Yeh, 1994[25]; Filigheddu et al., 2010[11]). ERBB/HER is a family of four transmembrane receptor tyrosine kinases including epidermal HER1/ERBB1 (also named as epidermal growth factor receptor), HER2/ ERBB2, HER3/ERBB3 and HER4/ ERBB4, couples binding of extracellular growth factor ligands to intracellular signaling pathways (Arteaga and Engelman, 2014[2]), and involved in different essential endometriosis related cellular processes such as proliferation, differentiation, cell motility, migration, adhesion and survival (Figure 5(Fig. 5)) (Kanehisa Laboratories, 2016[29]). Axon guidance is one of the novel pathways found to be familiar to endometriosis (Wei et al., 2015[52]). It was deduced that axon guidance is one of the strongly predicted pathways to be relevant for endometriosis. Although it may seem that axon guidance is not related to the endometriosis pathology, however it was found that nerves and blood vessels are closely related physically and in their morphogenesis and that there are common molecules involved in both axon guidance and angiogenesis (Filigheddu et al., 2010[11]). 

## Conclusion

The stressful micro milieu of serum derived from severe endometriosis cases can induce significant epigenetic changes in miRNA expression level in endometrial mesenchymal stem/stromal cells derived from non-endometriotic donors. This in turn affects their target genes that may play major roles in modulating the stem cell functions and fate. This *in vitro* proposed scenario may resemble the *in vivo* one, supporting MSC relevance to disease progression. Our findings provide new insights for miRNA based therapies targeting eMSCs of women affected by this debilitating condition.

## Acknowledgements

The authors would like to dedicate this work to the soul of prof. Wael El-Garaf. Fund of this study was provided by National Research Centre, Egypt. 

## Conflict of interest

The authors have no conflict of interest.

## Figures and Tables

**Table 1:**
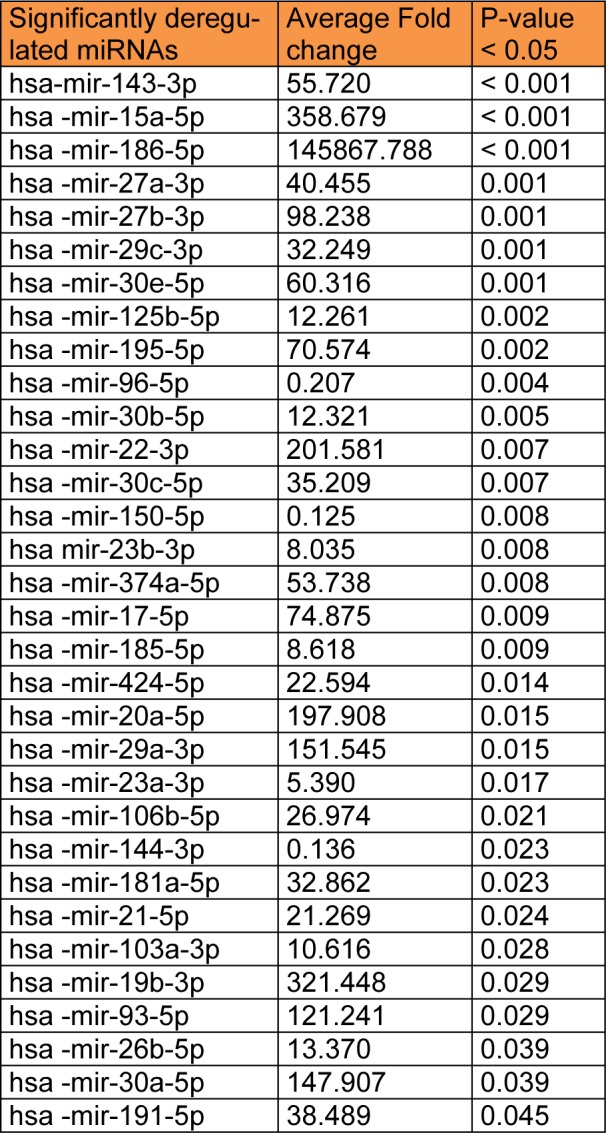
Significantly differentially expressed miRNAs

**Table 2:**
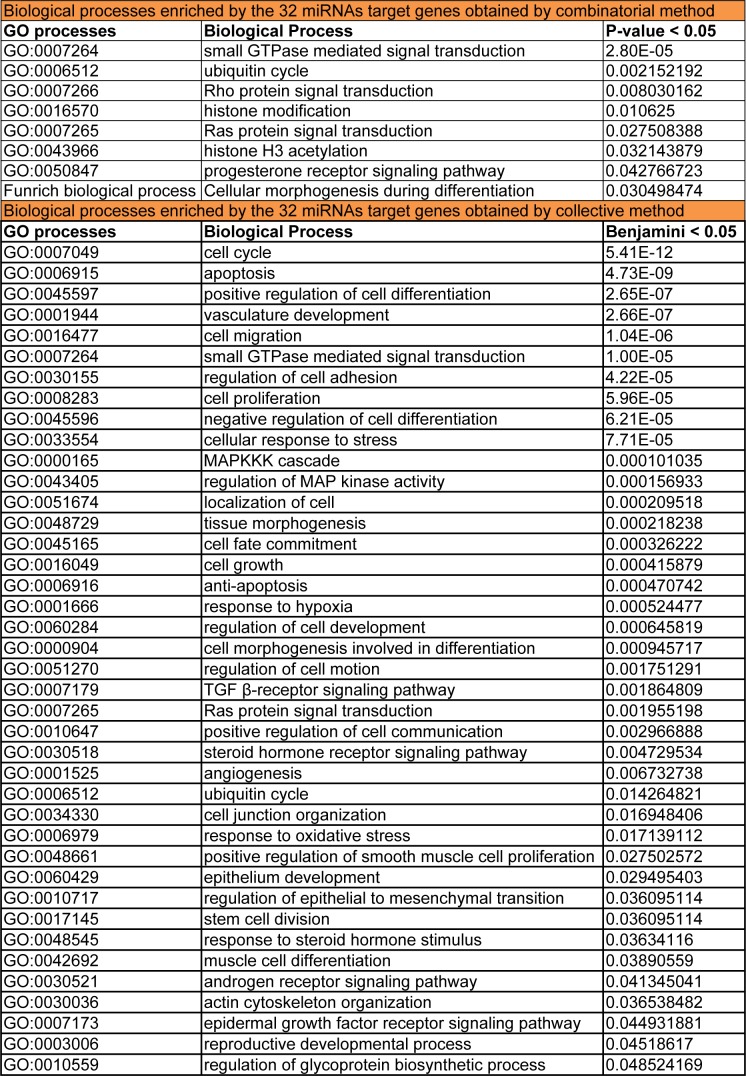
Enriched endometriosis related biological processes regulated by gene targets of the 32 differentially expressed miRNAs

## References

[1] Abe W, Nasu K, Nakada C, Kawano Y, Moriyama M, Narahara H (2013). miR-196b targets c-myc and Bcl-2 expression, inhibits proliferation and induces apoptosis in endometriotic stromal cells. Hum Reprod.

[2] Arteaga CL, Engelman JA (2014). ERBB receptors: from oncogene discovery to basic science to mechanism-based cancer therapeutics. Cancer Cell.

[3] Azmy O, Said K, El-Nouri MA, Elkady M, Mostafa M, Salama S (2014). Multicenter cohort molecular evidence of the presence of endometriosis-inducing factor (mir-130a) as a potent regulator of gene expression in endometriosis. Med Res J.

[4] Azmy OM, Elgarf WT (2012). MiRNA-130a, a potential endometriosis-inducing factor. Med Res J.

[5] Aznaurova YB, Zhumataev MB, Roberts TK, Aliper AM, Zhavoronkov AA (2014). Molecular aspects of development and regulation of endometriosis. Reprod Biol Endocrinol.

[6] Borghese B, Vaiman D, Mondon F, Mbaye M, Anaf V, Noel JC (2010). Gynecol Obstetr Fertil.

[7] Burney RO, Giudice LC (2012). Pathogenesis and pathophysiology of endometriosis. Fertil Steril.

[8] Choi J, Jo M, Lee E, Kim HJ, Choi D (2014). Differential induction of autophagy by mTOR is associated with abnormal apoptosis in ovarian endometriotic cysts. Mol Hum Reprod.

[9] Dennis G, Sherman BT, Hosack DA, Yang J, Gao W, Lane HC (2003). DAVID: Database for Annotation, Visualization, and Integrated Discovery. Genome Biol.

[10] Donnez J, Smoes P, Gillerot S, Casanas-Roux F, Nisolle M (1998). Vascular endothelial growth factor (VEGF) in endometriosis. Hum Reprod.

[11] Filigheddu N, Gregnanin I, Porporato PE, Surico D, Perego B, Galli L (2010). Differential expression of microRNAs between eutopic and ectopic endometrium in ovarian endometriosis. J Biomed Biotechnol.

[12] Finas D, Huszar M, Agic A, Dogan S, Kiefel H, Riedle S (2008). L1 cell adhesion molecule (L1CAM) as a pathogenetic factor in endometriosis. Hum Reprod.

[13] Gargett CE, Schwab KE, Deane JA (2016). Endometrial stem/progenitor cells: the first 10 years. Hum Reprod Update.

[14] Gargett CE, Schwab KE, Zillwood RM, Nguyen HP, Wu D (2009). Isolation and culture of epithelial progenitors and mesenchymal stem cells from human endometrium. Biol Reprod.

[15] Gilabert-Estelles J, Braza-Boils A, Ramon LA, Zorio E, Medina P, Espana F (2012). Role of microRNAs in gynecological pathology. Curr Med Chem.

[16] Giudice L, Leonardus J, Evers H, Healy DL (2012). Endometriosis: science and Practice.

[17] Giudice LC (2010). Clinical practice. Endometriosis. N Engl J Med.

[18] Goitre L, Trapani E, Trabalzini L, Retta SF (2014). The Ras superfamily of small GTPases: the unlocked secrets. Meth Mol Biol.

[19] Gupta S, Agarwal A, Krajcir N, Alvarez JG (2006). Role of oxidative stress in endometriosis. Reprod Biomed Online.

[20] Ha M, Kim VN (2014). Regulation of microRNA biogenesis. Nature Rev Mol Cell Biol.

[21] Hass R, Otte A (2012). Mesenchymal stem cells as all-round supporters in a normal and neoplastic microenvironment. Cell Commun Signal.

[22] Hawkins SM, Creighton CJ, Han DY, Zariff A, Anderson ML, Gunaratne PH (2011). Functional microRNA involved in endometriosis. Mol Endocrinol.

[23] Hsu CY, Hsieh TH, Tsai CF, Tsai HP, Chen HS, Chang Y (2014). miRNA-199a-5p regulates VEGFA in endometrial mesenchymal stem cells and contributes to the pathogenesis of endometriosis. J Pathol.

[24] Huang da W, Sherman BT, Lempicki RA (2009). Systematic and integrative analysis of large gene lists using DAVID bioinformatics resources. Nature Protoc.

[25] Huang JC, Yeh J (1994). Quantitative analysis of epidermal growth factor receptor gene expression in endometriosis. J Clin Endocrinol Metab.

[26] Ilad RS, Fleming SD, Bebington CR, Murphy CR (2004). Ubiquitin is associated with the survival of ectopic stromal cells in endometriosis. Reprod Biol Endocrinol.

[27] Jeong JW (2014). In search of molecular mechanisms in endometriosis. Endocrinology.

[28] Jiang Y, Jahagirdar BN, Reinhardt RL, Schwartz RE, Keene CD, Ortiz-Gonzalez XR (2002). Pluripotency of mesenchymal stem cells derived from adult marrow. Nature.

[29] Kanehisa Laboratories (2016). KEGG PATHWAY: ErbB signaling pathway - Homo sapiens (human).

[30] Kao AP, Wang KH, Chang CC, Lee JN, Long CY, Chen HS (2011). Comparative study of human eutopic and ectopic endometrial mesenchymal stem cells and the development of an in vivo endometriotic invasion model. Fertil Steril.

[31] Lin SC, Wang CC, Wu MH, Yang SH, Li YH, Tsai SJ (2012). Hypoxia-induced microRNA-20a expression increases ERK phosphorylation and angiogenic gene expression in endometriotic stromal cells. J Clin Endocrinol Metab.

[32] Livak KJ, Schmittgen TD (2001). Analysis of relative gene expression data using real-time quantitative PCR and the 2(-Delta Delta C(T)) method. Methods (San Diego, Calif).

[33] Macfarlane LA, Murphy PR (2010). MicroRNA: biogenesis, function and role in cancer. Curr Genom.

[34] Mari-Alexandre J, Sanchez-Izquierdo D, Gilabert-Estelles J, Barcelo-Molina M, Braza-Boils A, Sandoval J (2016). miRNAs Regulation and Its Role as Biomarkers in Endometriosis. Int J Mol Sci.

[35] Maruyama T (2014). Endometrial stem/progenitor cells. J Obstetr Gynaecol Res.

[36] Mathieu J, Ruohola-Baker H (2013). Regulation of stem cell populations by microRNAs. Adv Exp Med Biol.

[37] Matsuzaki S, Botchorishvili R, Pouly JL, Canis M (2014). Targeting the Wnt/beta-catenin pathway in endometriosis: a potentially effective approach for treatment and prevention. Mol Cell Ther.

[38] Monteiro JB, Colon-Diaz M, Garcia M, Gutierrez S, Colon M, Seto E (2014). Endometriosis is characterized by a distinct pattern of histone 3 and histone 4 lysine modifications. Reprod Sci.

[39] Mu L, Zheng W, Wang L, Chen XJ, Zhang X, Yang JH (2008). Alteration of focal adhesion kinase expression in eutopic endometrium of women with endometriosis. Fertil Steril.

[40] Ohlsson Teague EM, Van der Hoek KH, Van der Hoek MB, Perry N, Wagaarachchi P, Robertson SA (2009). MicroRNA-regulated pathways associated with endometriosis. Mol Endocrinol.

[41] Okamoto M, Nasu K, Abe W, Aoyagi Y, Kawano Y, Kai K (2015). Enhanced miR-210 expression promotes the pathogenesis of endometriosis through activation of signal transducer and activator of transcription 3. Hum Reprod.

[42] OuYang Z, Hirota Y, Osuga Y, Hamasaki K, Hasegawa A, Tajima T (2008). Interleukin-4 stimulates proliferation of endometriotic stromal cells. Am J Pathol.

[43] Pan Q, Luo X, Toloubeydokhti T, Chegini N (2007). The expression profile of micro-RNA in endometrium and endometriosis and the influence of ovarian steroids on their expression. Mol Hum Reprod.

[44] Pathan M, Keerthikumar S, Ang CS, Gangoda L, Quek CY, Williamson NA (2015). FunRich: An open access standalone functional enrichment and interaction network analysis tool. Proteomics.

[45] Rasheed K, Atta H, Taha T, Azmy O, Sabry D, Selim M (2010). A novel endometriosis inducing factor in women with endometriosis. J Stem Cells Regen Med.

[46] rASRM (1997). Revised American Society for Reproductive Medicine classification of endometriosis: 1996. Fertil Steril.

[47] Rocha AL, Reis FM, Taylor RN (2013). Angiogenesis and endometriosis. Obstetr Gynecol Int.

[48] Santulli P, Marcellin L, Tosti C, Chouzenoux S, Cerles O, Borghese B (2015). MAP kinases and the inflammatory signaling cascade as targets for the treatment of endometriosis?. Exp Opin Ther Targets.

[49] Sasson IE, Taylor HS (2008). Stem cells and the pathogenesis of endometriosis. Ann N Y Acad Sci.

[50] Sourial S, Tempest N (2014). Theories on the pathogenesis of endometriosis. Int J Reprod Med.

[51] Vasquez YM, Wu SP, Anderson ML, Hawkins SM, Creighton CJ, Ray M (2016). endometrial expression of steroidogenic factor 1 promotes cystic glandular morphogenesis. Mol Endocrinol.

[52] Wei S, Xu H, Kuang Y (2015). Systematic enrichment analysis of microRNA expression profiling studies in endometriosis. Iran J Basic Med Sci.

[53] Weng H, Liu F, Hu S, Li L, Wang Y (2014). GnRH agonists induce endometrial epithelial cell apoptosis via GRP78 down-regulation. J Translat Med.

[54] Wetendorf M, DeMayo FJ (2014). Progesterone receptor signaling in the initiation of pregnancy and preservation of a healthy uterus. Int J Develop Biol.

[55] Witz CA (2003). Cell adhesion molecules and endometriosis. Semin Reprod Med.

[56] Zhang H, Xue J, Li M, Zhao X, Wei D, Li C (2015). Metformin regulates stromal-epithelial cells communication via Wnt2/beta-catenin signaling in endometriosis. Mol Cell Endocrinol.

